# Design and application of an efficient cellulose-degrading microbial consortium and carboxymethyl cellulase production optimization

**DOI:** 10.3389/fmicb.2022.957444

**Published:** 2022-07-15

**Authors:** Guoyan Zhang, Yuanjie Dong

**Affiliations:** College of Resources and Environment, Shandong Agricultural University, Taian, China

**Keywords:** cellulose degrading strains, isolation, microbial consortium, condition optimization, rabbit feces, sesame oil cake, carboxymethyl cellulase

## Abstract

Microbial consortia with high cellulase activities can speed up the composting of agricultural wastes with high cellulose contents and promote the beneficial utilization of agricultural wastes. In this paper, rabbit feces and sesame oil cake were used as feedstocks for compost production. Cellulose-degrading microbial strains were isolated from compost samples taken at the different composting stages and screened *via* Congo red staining and filter paper degradation test. Seven strains, *Trichoderma reesei*, *Escherichia fergusonii*, *Proteus vulgaris*, *Aspergillus glaucus*, *Bacillus mycoides*, *Corynebacterium glutamicum*, and *Serratia marcescens*, with high activities of carboxymethyl cellulase (CMCase), filter paper cellulase (FPase), and β-glucosidase (β-Gase) were identified and selected for consortium design. Six microbial consortia were designed with these strains. Compared with the other five consortia, consortium VI composed of all seven strains displayed the highest cellulase activities, 141.89, 104.56, and 131.18 U/ml of CMCase, FPase, and β-Gase, respectively. The single factor approach and response surface method were employed to optimize CMCase production of consortium VI. The optimized conditions were: culture time 4.25 days, culture temperature 35.5°C, pH 6.6, and inoculum volume 5% (v/v). Under these optimized conditions, the CMCase activity of consortium VI was up to 170.83 U/ml. Fermentation experiment of rabbit feces was carried out by using the consortium VI cultured under the optimal conditions. It was found that the application effect was better than other treatments, and the fermentation efficiency and nutrient content of the pile were significantly improved. This study provides a basis for the design of microbial consortia for the composting of agricultural wastes with high cellulose contents and provides a support for beneficial utilization of agricultural wastes.

## Introduction

Cellulose, a carbohydrate polymer or polysaccharide composed of D-glucose units linked by β-1, 4 glycosidic bonds (Shaikh et al., 2013), is the most abundant organic polymer in nature and is considered to be an almost inexhaustible source of raw materials for various products (Eida et al., 2012). Cellulose is not only a major component of such agricultural wastes as crop straws but also a major component of the feces of herbivorous animals. For example, cellulose accounts for 30–50% of the dry weight of rabbit feces (Mesa et al., 2021). With the development of agriculture and economy, the generation of animal feces has been increasing in China. For example, more than 140 million tons of rabbit feces are produced annually (Mesa et al., 2021). Rabbit feces, one of the main by-products of the rabbit breeding industry, not only has a high cellulose content but also is rich in nutrients such as nitrogen, phosphorus, potassium, and organic carbon, with low heavy metal contents and suitable acidity (Li et al., 2013). In most cases, rabbit feces is applied to farmlands as manure or buried on site, which not only causes nutrient loss but also causes serious environmental pollution (Zhang et al., 2018). Using rabbit feces as feedstock to produce environmentally friendly, high-quality compost is a plausible way of proper disposal or utilization.

However, cellulose is of complex structure and high crystallinity (Kaur et al., 2021). Its decomposition is the rate-limiting step in compost production (Tuli et al., 2018). Cellulase can efficiently catalyze the decomposition of cellulose. Cellulase systems produced using microorganisms have long been applied in many fields such as food, paper, biofuel, and feed production (Gong et al., 2020; Agrawal et al., 2021). But low enzyme production, low enzyme activity, and poor adaptability of microbial strains have been the bottleneck in the large-scale production of cellulase. Therefore, the key to solving the problem is to screen efficient cellulase-producing microbial strains and design efficient microbial consortia. Many cellulase-producing microbial strains have been isolated from a variety of materials such as compost and cattle manure (Xi et al., 2015), vermicompost (Karthika et al., 2020), soil (Gong et al., 2020), and white wine lees (Yang et al., 2020). The cellulose-degrading bacterial strains mainly belong to *Bacillus* spp., *Streptococcus* spp., and *Pseudomonas* spp., the fungal strains mainly belong to *Trichoderma* spp., *Penicillium* spp., and *Aspergillus* spp., and the actinomycete strains mainly belong to *Streptomyces* spp. and *Nocardia* spp. (Agrawal et al., 2016).

Though many studies indicate the presence of various cellulase-producing microbial strains in rabbit feces (Dong et al., 2017; Montes-Carreto et al., 2021), few have isolated efficient cellulose-degrading strains from rabbit feces. Dong et al. (2017) isolated two Gram-positive cellulose-degrading Bacillus strains from rabbit feces, but their cellulose-degrading efficiency warrants further evaluation.

Single microbial strains are generally not very efficient in cellulose degradation, and increasing cellulase production through gene induction is not cost-effective. In contrast, microbial consortia composed of multiple microbial strains often show higher cellulase activities than single strains and thereby are attracting more and more attention. Gu et al. (2012) reported that the cellulase activity of a hemicellulose-degrading consortium was up to 299 U/ml. The six members of the consortium were isolated from rice field soils and rotten rice straws. Jiang et al. (2021) reported that the cellulose-degrading efficiency of a five-member fungal consortium was significantly higher than that of its individual members. However, when a consortium contains competing members, its cellulose-degrading efficiency may be inferior to that of individual members (Boddy, 2000). In addition, the members of a consortium as an individual require different conditions for their own maximum cellulase production. When they work as a team (consortium), it is very important that the conditions they share ensure maximum cellulase production of the consortium instead of an individual member.

Sesame oil cake is a by-product of sesame oil extraction. As of 2020, the annual generation of sesame oil cake in China had reached 370,000 tons. Sesame oil cake is rich in amino acids and is a rare protein resource with a protein content of higher than 50% (Cheng, 2017). Our preliminary study shows that when rabbit feces and sesame oil cake are used as feedstocks for compost production, the cellulose in rabbit feces undergoes rapid decomposition, and high-quality compost can be obtained in short periods (Sun et al., 2021). This indicates the presence of efficient cellulose-degrading microbial strains in the compost pile.

In this study, rabbit feces and sesame oil cake were used as feedstocks for compost production, and compost samples were taken at different composting stages for cellulose-degrading microbial strain isolation. Then, the isolates were identified and cellulose-degrading microbial consortia were designed. Finally, an efficient consortium was selected and the culture conditions were optimized for maximum cellulase production of the consortium. This study was aimed to solve the bottleneck in the large-scale production of cellulase and provide a technical basis for the production of high-quality organic fertilizers as well as the beneficial utilization of agricultural wastes.

## Materials and methods

### Reagents and compost feedstocks

The reagents used were of analytical grade and purchased from Shanghai Macleans Biochemical Technology Company unless otherwise stated. Feces of healthy adult rabbits was collected from a rabbit breeding farm near Tai’an City, Shandong Province, China. Sesame oil cake was taken from a small sesame oil factory in Tai’an City. Wood chips were purchased from the local building materials market and crushed for later use.

### Composting and sampling

Composting was carried out at the experimental station of the College of Resources and Environment, Shandong Agricultural University. Rabbit feces and sesame oil cake were mixed at 1:1 on a dry weight basis, and each barrel was composed of 4 kg rabbit feces and 4 kg sesame oil cake. Wood chips were added to adjust the ratio of carbon and nitrogen (C/N) to 25:1. The mixed rabbit feces had a moisture content at 45.19%, total nitrogen (TN) at 16.38 g/kg, total organic carbon (TOC) at 547.17 g/kg, pH at 6.87, ratio of carbon and nitrogen (C/N) at 33.37; Sesame oil cake had a moisture content at 8.56%, TN at 83.36 g/kg, TOC at 612.81 g/kg, C/N at 7.35; The wood chips had a moisture content at 10.36%, TN at 4.68 g/kg, TOC at 683.92 g/kg, C/N at 145.51. All the materials were mixed and loaded into polyethylene fermentation barrel. Water was added to the compost pile to adjust the moisture content to approximately 60%, and natural fermentation was carried out. Temperature in the compost pile was measured daily. At days 4 (heating stage), 10 (thermophilic stage), and 20 (cooling and maturity stage) of the 24-days composting process, after the compost pile was turned and thoroughly mixed, 30 g samples were collected at the 5-, 15-, and 20-cm depths, mixed, put in a sterilized polythene bag, immediately transported to the laboratory, and stored at 4°C.

### Isolation and screening of cellulose-degrading microbial strains

Microbial strains were isolated from compost samples using the dilution plate method. Briefly, 10 g compost sample was mixed with 90 ml sterile distilled water and shaken at 150 r/min for 30 min at the sampling temperature (i.e., the compost pile temperature when the sample was taken). 10-fold serial dilutions were prepared with sterilized distilled water, and then 150 μl 10^–3^–10^–7^ dilution was spread onto the carboxymethyl cellulose (CMC) isolation medium containing (g/L): CMC-Na 15.0, NH_4_NO_3_ 1.0, MgSO_4_⋅7H_2_O 0.5, KH_2_PO_4_ 1.0, yeast extract 1.0, and agar 20 in distilled water (Eida et al., 2012). The inoculated plates were incubated at the sampling temperatures for 3 days. The morphologically identified single colonies were selected with an inoculating needle and inoculated onto pH 7.0 CMC enrichment medium, which contained (g/L): CMC-Na 15.0, NH_4_NO_3_ 1.0, MgSO_4_⋅7H_2_O 0.5, KH_2_PO_4_ 1.0, NaCl 1.0, peptone 1.0, yeast extract 1.0, and agar 20 in distilled water. The enriched isolates were then preserved at −80°C (Zhang et al., 2018).

The enriched isolates were evaluated for their cellulase production by Congo red staining as described by Rawway et al. (2018). Briefly, each isolate was spotted on the CMC isolation medium with three spots per plate and incubated at the sampling temperature for 3 days. Then, the medium was flooded with 0.1% (weight/volume) Congo red reagent, allowed to stand for 30 min at room temperature, and washed with 1 mol/L NaCl. The formation of a clear zone of hydrolysis around a colony indicates cellulose degradation. The ratio of the clear zone diameter to colony diameter was used for cellulase production evaluation.

Microbial strains showing larger clear zone diameter to colony diameter ratios were further evaluated for their cellulolytic effect using filter paper degradation test. The isolates were first cultured in CMC liquid medium (CMC enrichment medium without agar) with shaking at 120 r/min for 3 days at the sampling temperatures. Then, they were inoculated to the pH 7.0 filter paper strip disintegration medium (containing KH_2_PO_4_ 1.0 g/L, NaCl 0.1 g/L, MgSO_4_⋅7H_2_O 0.5 g/L, NaNO_3_ 1.0 g/L, yeast extract 0.1 g/L, CaCl_2_ 0.1 g/L, and sterilized filter paper of 1 × 6 cm) and incubated with shaking at the sampling temperatures for 7 days. Isolates showing high cellulose-degrading ability in both the filter paper test and CMC isolation medium were selected for further analysis (Guder and Krishna, 2019).

### Determination of cellulase activity

For cellulase activity determination, the selected strains were cultured in pH 7.0 liquid enzyme production medium with shaking (120 r/min) at the sampling temperatures for 3 days. The medium contained (g/L): CMC-Na 5.0, (NH_4_)_2_SO_4_ 1.4, MgSO_4_⋅7H_2_O 0.3, KH_2_PO_4_ 2.0, CaCl_2_ 0.3, FeSO_4_⋅7H_2_O 0.005, MnSO_4_ 0.0016, ZnSO_4_⋅7H_2_O 0.0014, CoCl_2_ 0.002, and peptone 5 (Gong et al., 2020). Then, the broth was centrifuged at 5,000 r/min and 4°C for 10 min. The supernatant (i.e., crude enzyme extract) was collected for cellulase activity determination.

Cellulase activity was determined using the 3, 5-dinitrosalicylic acid method recommended by the International Union of Pure and Applied Chemistry (IUPAC) (Guder and Krishna, 2019). Carboxymethyl cellulase (CMCase) activity, filter paper cellulase (FPase) activity or total cellulase activity, and β-glucosidase (β-Gase) activity were determined by measuring the release of glucose from the substrates of CMC-Na, filter paper, and salicin, respectively (Zhang et al., 2018). One unit of cellulase activity is expressed as the quantity of enzyme required for the release of 1 μg glucose per milliliter substrate per minute under standard assay conditions (Rawway et al., 2018).

### Strain identification

Morphological, physiological, and biochemical identification of the strains were carried out according to the Manual of Systematic Identification of Common Bacteria and Berger’s Manual of Bacterial Identification (Bu and Ji, 1984; Dong and Cai, 2001). Gram staining and eosin methylene blue agar were used to differentiate bacteria and fungi, respectively. Strain morphology was observed with a microscope (LJ-JX2030, Songgang Longji instrument equipment company, Shenzhen, China).

Bacteria and actinomycetes were identified by 16S rRNA sequence alignment, and fungi were identified by the D1-D2 region of the large-subunit RNA gene (28S rDNA). The total DNA of bacteria and actinomycetes was extracted with the bacterial genome extraction kit (31516KC4, AXYGEN, Silicon Valley, CA, United States) and amplified with the universal primer pair 27F (5′-AGTTTGATCMTGGCTCAG-3′) and 1492R (5′-GGTTACCTTGTTACGACTT-3′) (Lane, 1991). Total fungal DNA was extracted with the fungal genomic DNA extraction kit (D3590-01, Omega, Norcross, GA, United States) and amplified with the primer pair NL1 (5′-GCATATCAATAAGCGGAGGAAAAG-3′) and NL4 (5′-GGTCCGTGTTTCAAGACGG-3′) (Kurtzman and Robnett, 1997). The cycling conditions were as follows: initial denaturation at 94°C for 3 min, 30 cycles of denaturation at 94°C for 30 s, primer annealing at 54°C for 30 s, and extension at 72°C for 90 s (fungal extension at 72°C for 45 s), and a final extension at 72°C for 10 min. The amplified products were electrophoresed on 1% agarose gel for purity, quantity, and size confirmation. The PCR products were sent to Nanchang Kechang Biotechnology Co., Ltd., Nanchang, China for sequencing. The resulting sequences were compared with those in the National Center for Biotechnology Information (NCBI) GenBank using the blastn program. Phylogenetic trees were constructed with the neighbor-joining method using the MEGA7.0 software, and Bootstrap was used to verify the credibility of the calculated branch of the phylogenetic tree (Zhang et al., 2018).

### Design of cellulose-degrading microbial consortia

**FIGURE 1:**
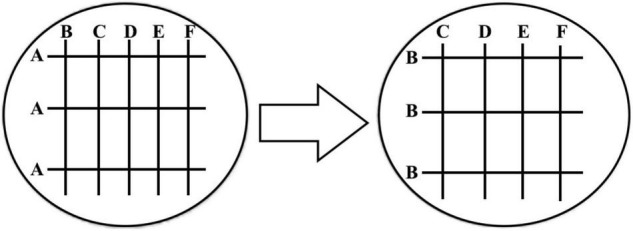
Schematic diagram showing the layout of strain inoculation on plates in the antagonism test. In this figure, A represents a random dominant strain and BCDEF, etc. represent other different dominant strains. After streaking on the plate, we observed whether there was a weak or non-growing antagonistic phenomenon at the intersection of A strain and BCDEF, etc. strain, and if it grew well, there was no antagonistic effect. Further, we observed whether strain B and strain CDEF, etc. were antagonistic, and so on.

To test the antagonism between the selected efficient cellulose-degrading strains, they were inoculated on CMC enrichment medium (containing CMC-Na 15.0 g/L, NH_4_NO_3_ 1.0 g/L, MgSO_4_⋅7H_2_O 0.5 g/L, KH_2_PO_4_ 1.0 g/L, NaCl 1.0 g/L, peptone 1.0 g/L, yeast extract 1.0 g/L, and agar 20 g/L in distilled water) as shown in Figure 1 and incubated for 3 days (the strains selected from the same stage were cultured at the sampling temperature, while the strains selected from multiple stages were cultured at 30°C suitable for most strains). The appearance of an inhibition zone between two strains indicates antagonism. Strains that were not antagonistic to each other were selected for the design of cellulose-degrading microbial consortia (Liu, 2019).

For the design of cellulose-degrading microbial consortia, the non-antagonistic strains were randomly selected and mixed in equal volumes (v/v). The consortia were cultured in liquid enzyme production medium and incubated with shaking (120 r/min) at the sampling temperature for 3 days. After that, the activities of CMCase, FPase, and β-Gase were determined, and the consortium with the highest cellulase activities was selected for culture condition optimization (Liu, 2019).

### Culture condition optimization for cellulase production

Four influencing factors, including culture time, culture temperature, pH, and inoculum volume, were tested in sequence with single factor experiments to optimize the culture conditions for cellulase production of the selected consortium. The optimal value of a factor obtained in an experiment would be used in the subsequent experiments, and the general culture conditions were: 30°C, pH 7, and inoculum volume of 5% (v/v). Culture times of 1, 2, 3, 4, 5, 6, and 7 days, culture temperatures of 25, 30, 35, 40, 45, and 50°C, pHs of 5.0, 5.5, 6.0, 6.5, 7.0, 7.5, and 8.0, and inoculum volumes of 1, 3, 5, 7, 9, and 11% (v/v) were tested (Liu, 2019; Gong et al., 2020).

Based on the results of single factor experiments, the Box–Behnken design of response surface methodology (RSM) was used to further optimize the influencing factors of cellulase production of the consortium using the Design-Expert software (version 11.0, StatEase Inc., Minneapolis, MS, United States). Multiple regression was fitted to the experimental data to obtain the optimal values of the factors (Gong et al., 2020).

### Verification of aerobic composting of consortium VI

The composting experiment was carried out for 26 days in a self-built, aerated static composting boxes, and the composting method was the same as the Section “composting and sampling.” The mixed rabbit feces had a moisture content at 68.82%, total nitrogen (TN) at 21.58 g/kg, TOC at 363.08 g/kg, pH at 6.95, ratio of carbon and nitrogen (C/N) at 16.83; Sesame oil cake had a moisture content at 11.62%, TN at 69.16 g/kg, TOC at 619.99 g/kg, C/N at 8.97. And the wood chips were also added to improve compost texture and adjust the ratio of carbon to nitrogen. The wood chips had a moisture content at 12.74%, TN at 4.30 g/kg, TOC at 607.08 g/kg, C/N at 141.13. The moisture content of the final mixed material was 52.61%. Three treatments were designed: the addition of consortium VI which cultured under the conditions of optimal enzyme production (CR), the addition of commercial agent EM (EM) and the addition of sterile medium as the control group (CK). The EM microbial agent was purchased from Shandong Nuojie Biotechnology Co., Ltd. (Shandong, China) composed of dozens of microorganisms belonging to 5 families and 10 genera. The main components were yeast, photosynthetic bacteria and lactic acid bacteria, etc. It was a dark brown liquid with 10 × 10^8^ cfu/ml and possessed a slight smell of fermentation. The inoculation mass percentage of bacterial liquid treated with CR and EM to the fresh weight of the pile was 0.5% (w/w), and the concentrations of the strain were controlled to 2.0∼2.11 × 10^8^ cfu/ml by the plate colony counting method (Chinese Agricultural Standard of Organic Fertilizer NY884-2012), so as to avoid the influence of different viable bacteria on composting. The composting materials were stacked inside high-density polyethylene boxes, each having volume of 100 L. The boxes were modified to allow air movement. The aeration rate was controlled at 120 ml/min by a flow meter during the composting process. The exhaust gas was passed through an Erlenmeyer flask containing 1 L of 1 mol/L NaOH, and the carbon dioxide was captured. To monitor the aerobic composting process, the following parameters were determined: the organic matter and total humic acid content, the cumulative emission of carbon dioxide, total nitrogen content, C/N ratio and the seed germination index (GI). The measurement of organic matter was according to the Chinese Agricultural Standard of Organic Fertilizer NY525-2012. The total humic acid was determined according to the method outlined by Sun et al. (2021). The carbon dioxide was trapped by a sodium hydroxide solution and measured by titration. The samples were digested with H_2_SO_4_/H_2_O_2_ and N content was determined by the Kjeldahl (1883) method. The GI was determined using Chinese pakchoi seeds, with a 1:10 solid-water suspension.

### Statistical analysis

The ggplot2 package in R (version 3.2.1) was used for figure plotting. Data were subjected to analysis of variance (ANOVA). The LSD.test function of agricolae package in R was used for mean separation. Adobe illustrator CC 2014 software was used for vector graphics processing.

## Results

### Strain isolation and screening

**FIGURE 2:**
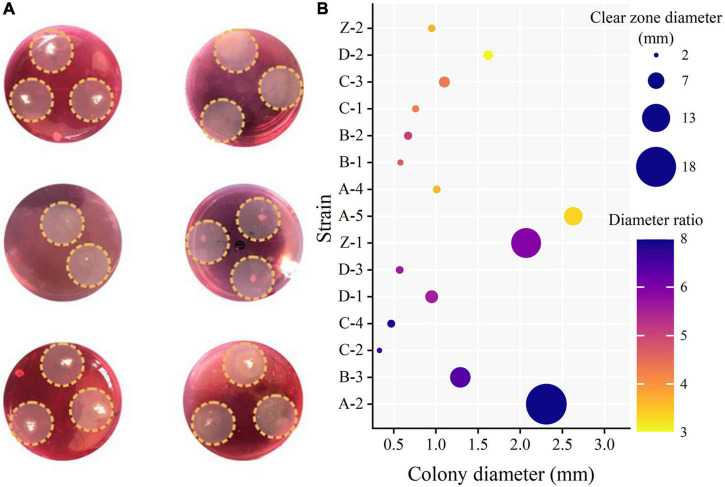
Photos showing Congo red staining results **(A)** and clear zone diameters, colony diameters, and ratios of clear zone diameters and colony diameters **(B)** of some strains. **(A)** Congo red staining of some strains. **(B)** Congo red stained clear zone size.

**FIGURE 3:**
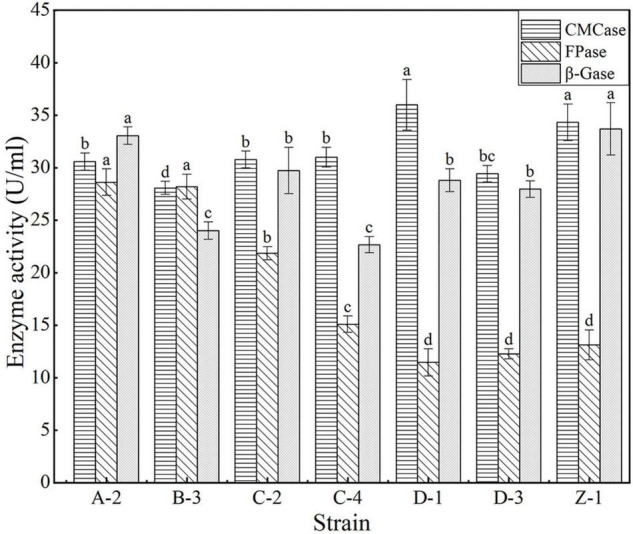
Activities of cellulase, including carboxymethyl cellulase (CMCase), filter paper cellulase (FPase), and β-glucosidase (β-Gase), of the seven cellulose-degrading bacterial strains. Different letters indicate significant differences between strains for a same enzyme (*P* < 0.05).

A total of 43 cellulose-degrading microbial strains were isolated from the compost samples collected at different composting stages, of which 15 produced clear zones on the CMC agar plates after Congo red staining (Figure 2A). In particular, the strains A-2, B-3, C-2, C-4, D-1, D-3, and Z-1 produced larger clear zones than the other strains (Figure 2B), and the ratio of the clear zone diameter to colony diameter reached 5.87–7.73. Congo red reacts with cellulose to form a red compound. If a strain produces cellulase, the cellulose in the medium would be decomposed, resulting in a clear zone around the colony after Congo red staining. The larger the ratio of the clear zone diameter to colony diameter, the greater the cellulase-producing capacity of the strain. However, some studies showed that the ratio of the clear zone diameter to colony diameter correlated poorly with the cellulase-producing capacity of a microbial strain (Guder and Krishna, 2019; Gong et al., 2020). Therefore, filter paper degradation test was performed to further verify the cellulase-producing capacity of the 15 strains (Wang et al., 2015). The results showed that the filter paper strips in the media inoculated with strains A-2, B-3, C-2, C-4, D-1, D-3, or Z-1 were completely degraded within 7 days (Table 1), verifying the cellulase-producing capacity of these seven strains. Of these seven strains, A-2 showed the highest cellulase activity, with a total activity of 92.22 U/ml for the three cellulases (Figure 3).

**TABLE 1:** Results of the filter paper strip decomposition experiment.

Time	Strain														

	**A-2**	**A-4**	**A-5**	**B-1**	**B-2**	**B-3**	**C-1**	**C-2**	**C-3**	**C-4**	**D-1**	**D-2**	**D-3**	**Z-1**	**Z-2**
Day 1	**+**			**+**		**+**								**+**	**+**
Day 2	**+**		**+**	**+**		**+**				**+**	**+**	z	**+**	**++**	**++**
Day 3	**+**		**+**	**+**		**++**		**+**		**+**	**+**	**+**	**+**	**++**	**++**
Day 4	**++**		**+**	**+**		**++**	**+**	**+**		**++**	**++**	**+**	**++**	**+++**	**++**
Day 5	**++**	**+**	**+**	**+**	**+**	**+++**	**+**	**++**		**++**	**++**	**++**	**+++**	**+++**	**++**
Day 6	**++**	**+**	**+**	**+**	**+**	**+++**	**++**	**++**	**+**	**+++**	**++**	**++**	**+++**	**++++**	**++**
Day 7	**+++**	**+**	**++**	**++**	**+**	**++++**	**++**	**++**	**+**	**+++**	**+++**	**++**	**+++**	**++++**	**+++**

+ indicates that the edge of the filter paper was slightly swelled. ++ indicates that the whole filter paper strip was expanded and bent. +++ indicates that the shape of the filter paper strip was no longer recognizable. ++++ indicates that the filter paper strip had been decomposed into paste. A-2, A-4, A-5, B-1, B-2, B-3, C-1, C-2, C-3, C-4, D-1, D-2, D-3, Z-1, and Z-2 were different strains.

### Strain identification

**FIGURE 4:**
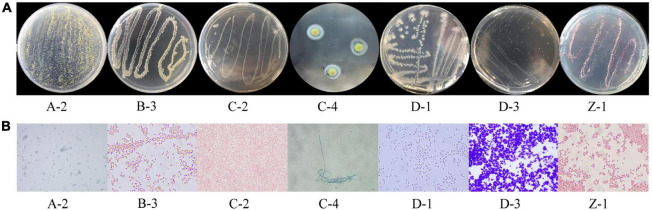
Colony and cell morphologies of the seven isolated cellulase-producing strains. **(A)** Colony morphology. **(B)** Cell morphology.

The colonies of strain A-2 showed floc morphology, with dense branched yellow-green aerial mycelia (Figure 4A). The colonies of strain B-3 were moist, slimy, and yellow. The colonies of strain C-2 were creamy white, flat, and irregular, with a putrid smell. Strain C-4 grew mildly, and its colonies showed raised yellow-green centers and fringed white edges. The colonies of strain D-1 had a special pseudo-root structure, flat and opaque, with a diffuse growth. The colonies of strain D-3 were milky white with a smooth surface. The colonies of strain Z-1 showed raised centers and were opaque, smooth, adhesive, and white at the early stage but red at the later stage. The cells of strains B-3, C-2, D-1, D-3, and Z-1 were rod-shaped, approximately 0.5–5 μm in length and 0.4–3 μm in width (Figure 4B). The cells of strains A-2 and C-4 were markedly longer than those of bacilli, approximately 20 μm long or longer, which endowed easy observation compared with the other strains. Gram staining results showed that strains D-1 and D-3 were positive, whereas strains B-3, C-2, and Z-1 were negative. The physiological and biochemical characteristics of the strains are shown in Table 2. Based on the Berger’s Manual of Bacterial Identification and the Manual of Fungal Identification, the preliminary judgment was that B-3 belongs to the genus *Escherichia* of Enterobacteriaceae, C-2 and Z-1 belong to the genera *Proteus* and *Serratia*, respectively, D-1 belongs to *Bacillus*, D-3 may belong to *Corynebacterium* or *Bifidobacterium*, and A-2 and C-4 belong to *Trichoderma* and *Aspergillus*, respectively.

**TABLE 2:** Physiological and biochemical characteristics of the seven isolated cellulase-producing strains.

Test item	Strain							

	**A-2**	**B-3**	**C-2**	**C-4**	**D-1**	**D-3**	**Z-1**
Nitrate reduction	−	−	−	+	+	+	+
Splitting of urea	−	+	−	−	−	−	−
Gelatin liquefaction	−	−	+	+	−	−	+
Hydrogen sulfide test	−	+	+	−	+	−	+
Amylase	+	−	−	+	+	−	−
Catalase	+	+	+	+	+	+	+
Indolephenol test	−	+	+	−	−	−	−
MR test	+	−	+	+	+	+	−
V-P test	−	+	+	−	−	−	+
Glucogenic acid	−	−	+	+	+	−	+
Glucose gas production	+	−	+	+	−	+	+

+positive reaction; −negative reaction.

**FIGURE 5:**
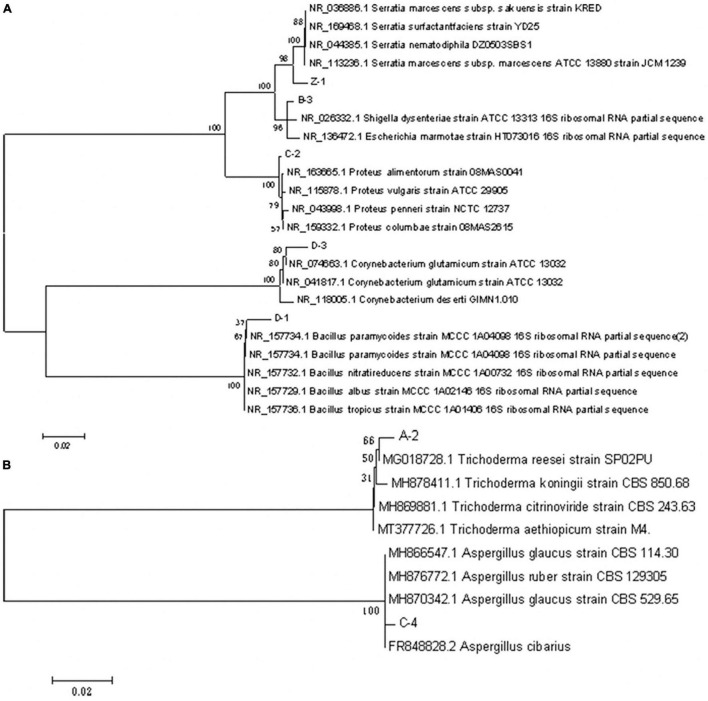
Phylogenetic trees of the seven isolated cellulase-producing microbial strains based on 16S rDNA (bacteria) and D1/D2 domains of the 28S rDNA (fungi) sequences. **(A)** Bacteria. **(B)** Fungi.

The 16S rDNA (bacteria) and D1/D2 domains of the 28S rDNA (fungi) sequences of the seven strains were submitted to GenBank for blastn comparison (Table 3). Based on the morphological, physiological, and biochemical characterization and phylogenetic analyses (Figure 5), strains A-2, B-3, C-2, C-4, D-1, D-3, and Z-1 were identified as *Trichoderma reesei*, *Escherichia marmotae*, *Proteus alimentorum*, *Aspergillus glaucus*, *Bacillus paramycoldes*, *Corynebacterium glutamicum*, and *Serratia marcescens* subsp., respectively. The sequence data determined in this study were submitted to GenBank and published with the following accession numbers: ON849069 (Z-1), ON849070 (D-1), ON849071 (D-3), ON849072 (C-2), ON849073 (B-3), ON849089 (C-4), ON849090 (A-2).

**TABLE 3:** Comparison results of strain similarity.

Strain	Closest relative	Similarity (%)
A-2	*Trichoderma reesei* strain SP02PU (MG018728.1)	99.83
B-3	*Escherichia marmotae* strain HT073016 (NR_136472.1)	99.02
C-2	*Proteus alimentorum* strain 08MAS0041 (NR_163665.1)	99.64
C-4	*Aspergillus glaucus* strain CBS 529.65 (MH870342.1)	99.12
D-1	*Bacillus paramycoldes* strain MCCC 1A04098 (NR_157734.1)	98.69
D-3	*Corynebacterium glutamicum* strain ATCC 13032 (NR_074663.1)	98.46
Z-1	*Serratia marcescens* subsp. Marcescens ATCC 13880 (NR_113236.1)	98.33

A-2, B-3, C-2, C-4, D-1, D-3, and Z-1 indicate the isolated cellulase-producing strains.

### Design of consortia

**FIGURE 6:**
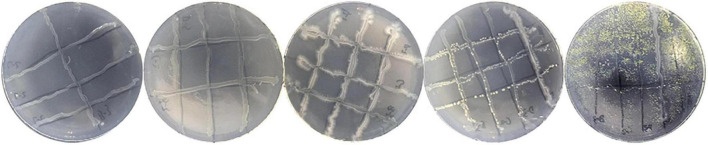
Photos showing the results of the antagonism test between the seven cellulase-producing strains.

The antagonism test showed that there was no antagonism between the seven strains (Figure 6). Therefore, any two of the seven strains can be used together in a consortium. Consortia I, II, III, IV, V, and VI were two-, three-, four-, five-, six-, and seven-member microbial consortia, respectively, designed *via* random combination of strains from the seven isolates. The six consortia showed different cellulase activities (Table 4). Consortia composed of more members displayed a higher total cellulase activity. The seven-member consortium VI had the highest total cellulase activity, with activities of CMCase, FPase, and β-Gase being higher than 100 U/ml, much higher than those of the other consortia. Compared with strain A-2, which, of the seven strains, had the highest cellulase activities, the activities of CMCase, FPase, and β-Gase of consortium VI increased by 364, 265, and 296%, respectively. The total cellulase activities of consortia II, III, IV, and V were also increased compared with any of their members, demonstrating that a microbial consortium composed of non-antagonistic strains can achieve higher cellulase activity than its members. However, the cellulase activity of consortium I was lower than that of its member strain A-2, with the activities of CMCase, FPase, and β-Gase lower by 7.25, 19.28, and 32.96%, respectively, indicating that the cellulase activity of a consortium may not be always higher than that of its members. This is because the strains composing a consortium may be very different in their vital activity and metabolism. One member may have an inhibitory effect on the other even though they are not antagonistic, lowering the cellulase production of the consortium as a whole. In addition, the strains were isolated from compost samples taken from different composting stages. They required different environmental conditions such as temperature and pH for optimal growth and cellulase production. When composing a consortium, they were subjected to the same environmental conditions, which may not be the optimal conditions for some members, leading to suboptimal growth and in turn lower cellulase production of these strains. Therefore, the cellulase activity of a microbial consortium may be lower than the sum of the cellulase activities of its members when they are cultured separately. Of the six consortia, consortium VI displayed the highest cellulase activities, and it underwent the subsequent optimization experiments so as to achieve higher cellulase production.

**TABLE 4:** Activities (U/ml) of CMCase, FPase, and β-Gase of the six consortia with different combinations of the isolated strains.

Consortium no.	Number of members	CMCase	FPase	β -Gase
I	2	28.35 ± 0.79	23.09 ± 2.36	22.16 ± 1.25
II	3	47.74 ± 0.94	49.41 ± 0.79	51.49 ± 1.12
III	4	68.78 ± 1.47	69.82 ± 0.65	72.01 ± 1.47
IV	5	89.48 ± 1.30	74.50 ± 1.10	82.61 ± 1.88
V	6	127.13 ± 1.72	81.47 ± 0.83	107.89 ± 1.10
VI	7	141.89 ± 1.41	104.56 ± 1.74	131.18 ± 1.26

### Single factor optimization

**FIGURE 7:**
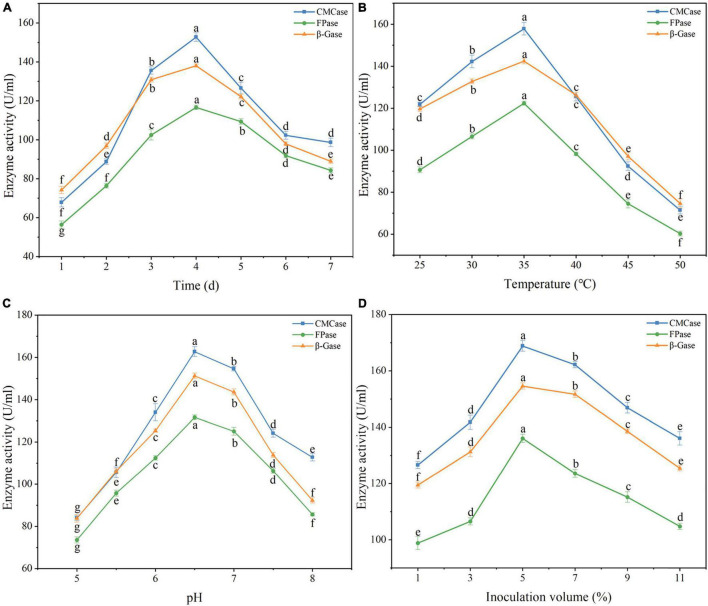
Optimization of culture conditions for cellulase production by microbial consortium VI by the single factor approach. In Panel **(A)**, the temperature was 30°C, the pH was 7, and the inoculum volume was 5% (v/v); in Panel **(B)**, the culture time was 4 days, the pH was 7, and the inoculum volume was 5% (v/v); in Panel **(C)**, the temperature was 35°C, the culture time was 4 days, and the inoculum volume was 5% (v/v); in Panel **(D)**, the temperature was 35°C, the culture time was 4 days, and the pH was 6.5. **(A)** Effect of time on enzyme production by consortium. **(B)** Effect of temperature on enzyme production by consortium. **(C)** Effect of initial pH on enzyme production by consortium. **(D)** Effect of inoculation volume on enzyme production by consortium. Error bars represent standard deviation; they were calculated using data from 3 technical replicates. Different letters (e.g., “a,” “b,” “c,” and “d”) represented significant difference (*p* < 0.05).

As shown in Figure 7A, culture time had a great influence on the activities of CMCase, FPase, and β-Gase. Maximum enzyme activities were achieved when culture time was 4 days, and lower enzyme activities were obtained when culture time was shorter or longer. The lower enzyme activities at shorter culture time are probably because it took the strains some time to get used to the culture environment. The lower enzyme activities at longer culture time may be due to nutrient deficiency. When the temperature was higher than 40°C, the enzyme activities decreased significantly (Figure 7B). The explanation may be that high temperature not only directly inhibits enzyme activity but also indirectly inhibits enzyme activity by negatively influencing microbial activity. When the temperature was 35°C, all enzyme activities displayed their maximum values, which were 10.97% (CMCase), 14.84% (FPase), and 7.29% (β-Gase) higher compared with those at 30°C. As shown in Figure 7C, with the increase of pH, the enzyme activities increased rapidly, peaked at pH 6.5, and then decreased. Similar result was reported by Mao et al. (2019), who isolated an efficient cellulose-degrading *Bacillus pumilus* strain from yak manure and found that the strain had maximum cellulase activity at pH 6.5. As shown in Figure 7D, with the increase of inoculum volume, the activities of the three enzymes first increased and then decreased, with the maximum enzyme activities achieved at the inoculum volume of 5% (v/v). The reason for the decreased enzyme activities at higher inoculum volumes may be because the oxygen and nutrients in the medium had been rapidly consumed, which affected the growth of the consortium and in turn the production of enzymes.

### Response surface optimization

Taking into account the interactions of the four factors, cellulase production was further optimized using RSM, with the activity of CMCase, a key cellulase in cellulose degradation, as the dependent variable *Y* and the four factors, i.e., culture time (*A*), culture temperature (*B*), pH (*C*), and inoculum volume (*D*), as the independent variables. Based on the Box–Behnken experimental design, a four-factor, three-level response surface design was adopted as showed in Table 5, and the results are shown in Table 6.

**TABLE 5:** Response surface test factors and levels.

Level	*A* culture time (d)	*B* temperature (°C)	*C* pH	*D* inoculum volume (%)
−1	3	30	6.0	3
0	4	35	6.5	5
1	5	40	7.0	7

A, culture time; B, temperature; C, pH; D, inoculum volume. They are four factors in response surface test, as the independent variables.

**TABLE 6:** Box–Behnken experimental design for the response surface test and results.

Test no.	*A* culture time	*B* temperature	*C* pH	*D* inoculum volume	*Y* CMCase activity (U/ml)
1	1	1	0	0	153.937
2	1	0	0	−1	153.537
3	1	0	0	1	156.189
4	1	0	1	0	155.426
5	1	0	−1	0	153.327
6	1	−1	0	0	157.563
7	0	0	0	0	169.755
8	0	0	−1	−1	137.281
9	0	0	0	0	170.556
10	0	0	0	0	168.324
11	0	0	−1	1	150.350
12	0	1	0	1	152.583
13	0	0	0	0	167.713
14	0	0	1	−1	151.514
15	0	1	1	0	158.078
16	0	−1	1	0	149.530
17	0	−1	0	1	149.091
18	0	0	1	1	150.217
19	0	1	0	−1	151.800
20	0	0	0	0	167.713
21	0	−1	−1	0	145.943
22	0	−1	−1	−1	133.655
23	0	1	−1	0	147.279
24	−1	0	−1	0	143.234
25	−1	−1	0	0	140.219
26	−1	0	1	0	152.182
27	−1	0	0	−1	143.691
28	−1	1	0	0	148.881
29	−1	0	0	1	145.714

A, culture time; B, temperature; C, pH; D, inoculum volume. They are four factors in response surface test, as the independent variables. Y indicates CMCase activity that is the response value in response surface test, as the dependent variable.

Multiple regression using the Design-Expert 10.0.3 software generated the following multiple regression model: *Y* = 168.635 + 4.67142*A* + 1.90683*B* + 3.14686 *C* + 1.58247*D* − 3.07188*AB* − 1.71243*AC* + 0.15741*AD* + 2.24591 *BC* − 0.244533*BD* − 3.14896*CD* − 8.65433*A*^2^ − 8.86245*B*^2^ − 9.6 254*C*^2^ − 10.4795*D*^2^. The ANOVA results for the regression equation are shown in Table 7. It can be seen that the effects of the factors on CMCase activity of consortium VI were in the order of: *A* > *C* > *B* > *D*. In other words, of the four factors, culture time had the greatest effect on CMCase activity of consortium VI, whereas inoculum volume had the smallest effect, with pH and culture temperature having medium effects. The coefficient of determination (*R*^2^) of the model was 0.9794, indicating a very good fit of the model to the data. In addition, *R*^2^_*Adj*_ = 0.9588, which indicates that the model can explain 95.88% of the variation in CMCase activity (Wang et al., 2018), and it was close to the predicted correlation coefficient (Pred *R*^2^). These results indicate that the model fit the experimental data very well and can be used to analyze and predict optimal CMCase activity.

**TABLE 7:** Analysis of variance (ANOVA) for the response value quadratic model.

Source	Sum of squares	df	Mean square	*F* value	*P* value	Significance
Model	2430.64	14	173.62	47.53	<0.0001	***
*A*	261.87	1	261.87	71.70	<0.0001	***
*B*	41.55	1	41.55	11.38	0.0046	**
*C*	122.30	1	122.30	33.48	<0.0001	***
*D*	28.62	1	28.62	7.83	0.0142	*
*AB*	37.75	1	37.75	10.33	0.0062	**
*AC*	11.73	1	11.73	3.21	0.0948	
*AD*	0.099	1	0.099	0.027	0.8715	
*BC*	22.05	1	22.05	6.04	0.0277	*
*BD*	0.21	1	0.21	0.057	0.8149	
*CD*	43.35	1	43.35	11.87	0.0039	**
*A* ^2^	477.59	1	477.59	130.76	<0.0001	***
*B* ^2^	509.18	1	509.18	139.41	<0.0001	***
*C* ^2^	589.45	1	589.45	161.39	<0.0001	***
*D* ^2^	711.93	1	711.93	194.92	<0.0001	***
Residual	51.13	14	3.65			
Lack of fit	44.55	10	4.46	2.71	0.1748	
Pure error	6.58	4	1.65			
Cor total	2481.77	28				

*Significant at P < 0.05; **Significant at P < 0.01; ***Significant at P < 0.001. R^2^ = 0.9794, Adj R^2^ = 0.9588, Pred R^2^ = 0.8990. A, culture time; B, temperature; C, pH; D, inoculum volume. They are four factors in response surface test, as the independent variables. AB, interaction between culture time and temperature; AC, interaction between culture time and pH; AD, interaction between culture time and inoculum volume; BC, interaction between temperature and pH; BD, interaction between temperature and inoculum volume; CD, interaction between pH and inoculum volume. A^2^, The quadratic term of culture time; B^2^, The quadratic term of temperature; C^2^, The quadratic term of pH; D^2^, The quadratic term of inoculum volume.

**FIGURE 8:**
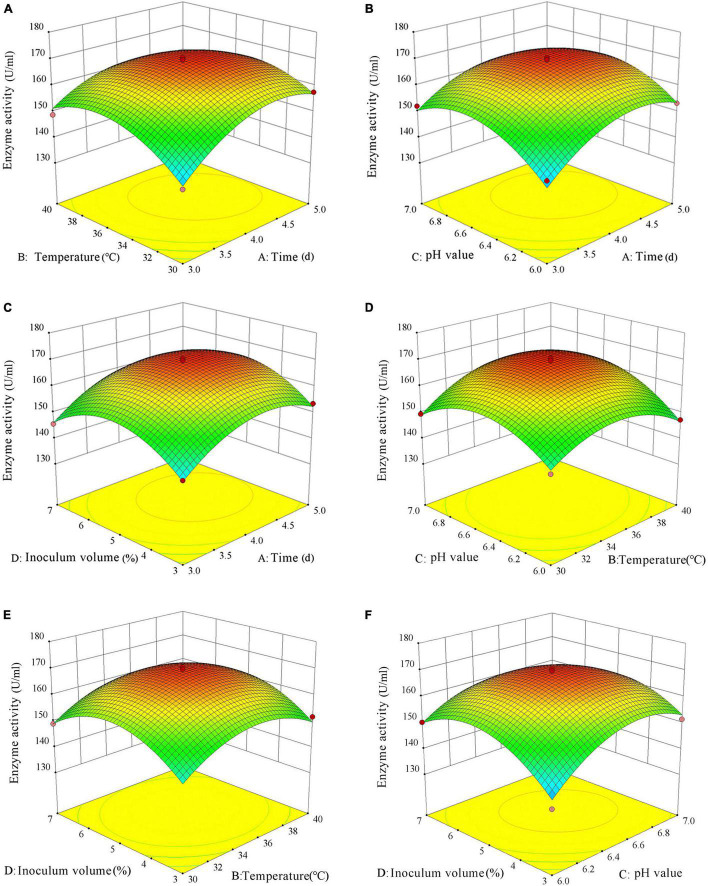
Response surface plots showing the interaction effects between factors on carboxymethyl cellulase (CMCase) activity of consortium VI. **(A)** The effect of the interaction between culture time and culture temperature on enzyme activity. **(B)** The effect of the interaction between culture time and pH on enzyme activity. **(C)** The effect of the interaction between culture time and inoculum volume on enzyme activity. **(D)** The effect of the interaction between culture temperature and pH on enzyme activity. **(E)** The effect of the interaction of culture temperature and inoculum volume on enzyme activity. **(F)** The effect of the interaction of pH and inoculum volume on enzyme activity. Different letters (e.g., “a,” “b,” “ab,” and “c”) in a same column indicate significant differences at *p* < 5%.

The effects of the interactions of the factors on CMCase activity are shown in Figure 8. It can be seen from Figure 8A that CMCase activity first increased and then decreased with increasing culture time or culture temperature. Maximum CMCase activity can be achieved at culture time of 4–4.5 days and culture temperature of 32–38°C. Similarly, the CMCase activity first increased and then decreased with the increase of culture time or pH (Figure 8B). Maximum CMCase activity can be obtained after 4–4.5-days culture at pH 6.4–6.8. As shown in Figure 8C, maximum CMCase activity can be obtained at culture time of 4–4.5 days and inoculum volume of 4–6% (v/v). It can be seen from Figure 8D that when the culture temperature was lower than 35°C, CMCase activity was positively correlated with culture temperature, but there was no such a relationship when culture temperature was higher than 35°C. Therefore, the optimal temperature for maximum CMCase activity would be close to 35°C. Similarly, the optimal pH for maximum CMCase activity was approximately 6.6. The subcircular shape of the contour of the interaction between culture temperature and inoculum volume indicates that the interaction between the two factors did not have a significant effect on CMCase activity (Figure 8E). The CMCase activity first increased and then decreased with increase in temperature or inoculum volume, peaking at culture temperature of 34–38°C and inoculum volume of 4–6% (v/v). As shown in Figure 8F, the interaction between pH and inoculum volume had a significant effect on CMCase activity. Maximum CMCase activity can be achieved at pH 6.4–6.8 and inoculum volume of 4–6% (v/v).

Then, Design-Expert 10.0.3 was used to further determine the optimal conditions for maximum CMCase activity. The results showed that the optimal conditions were: culture time 4.241 days, culture temperature 35.418°C, pH 6.572, and inoculum volume 5.110% (v/v). The predicted value of CMCase activity based on these conditions was 169.546 U/ml. To verify the accuracy of the above design results, an experiment with six replicates was performed, where the culture time was set to 4.25 days, the culture temperature was set to 35.5°C, the pH was set to 6.6, and the inoculum volume was set to 5% (v/v). The mean CMCase activity obtained was 170.83 ± 7.09 U/ml, very close to the predicted value, indicating that the established regression model can accurately reflect the effects of the four factors on CMCase activity, and that the optimal factor values obtained can be applied in the culture of consortium VI for maximum cellulase production.

The decomposition of cellulose requires the synergistic action of the three components, i.e., endoglucanase, exoglucanase, and β-glucosidase, of cellulase enzyme complex. It is very important that under the optimal culture conditions for maximum production of CMCase (endoglucanase), the production of FPase (exoglucanase), and β-Gase is also high, if not maximum as well. In this study, under the optimal culture conditions for maximum CMCase activity, FPase and β-Gase activities were 135.22 and 156.46 U/ml, respectively. Both FPase and β-Gase activities were even slightly higher than their maximum values obtained in the single factor optimization experiments. This result indicated that the optimal culture conditions for maximum CMCase activity obtained *via* RSM were favorable for FPase and β-Gase as well. This is of significance for the application of consortium VI.

### Verification results of aerobic composting

Table 8 presented the physicochemical properties of each treatment at the end of composting. Compared with CK, the organic matter content of each bacterial treatment agent was significantly decreased, while the CO_2_ accumulation and humic acid content were significantly increased. Compared with EM, the organic matter content was decreased by 17.78% in CR, CO_2_ accumulation was increased by 28.81%, and total humic acid content was increased by 12.60%. It indicated that the addition of consortium VI could significantly accelerate the degradation of organic matter, promote the formation of humic acid, and the effect of pile humification was the best. Compared with CK, the total nitrogen content of each bacterial treatment agent was increased, the C/N ratio was significantly decreased, and the GI was significantly increased (Table 8). Compared with EM, the total nitrogen content was increased by 4.12% in CR, C/N ratio was decreased by 21.21%, and the GI was increased by 19.85%. It indicated that the addition of consortium VI could significantly enhance the “concentration effect” of compost, improve the nutrient content, promote the maturity process, and reduce biological toxicity of compost products.

**TABLE 8:** Effects of different treatments on physicochemical properties (%) of composting.

Treatment	Organic matter	Total humic acid	CO_2_ cumulative emission	Total nitrogen	C/N	Germination index
CK	72.90 ± 1.59a	9.56 ± 0.29b	2.90 ± 0.27b	3.87 ± 0.18b	10.94 ± 0.45a	81.05 ± 0.05c
EM	66.95 ± 1.22b	12.31 ± 0.76a	3.01 ± 0.09b	4.13 ± 0.16ab	9.43 ± 0.54b	105.69 ± 0.08b
CR	55.05 ± 0.33c	13.86 ± 0.76a	3.88 ± 0.22a	4.30 ± 0.04a	7.43 ± 0.11c	126.67 ± 0.03a

CK, no bacterial agent; EM, EM commercial microbial agent; CR, consortium VI cultured under the optimal enzyme production conditions. Different letters in a same column indicate significant differences at P < 5%.

## Discussion

Using rabbit feces and sesame oil cake as feedstocks can obtain high-quality compost in short periods, which has a total nutrient content of more than 10% and plant growth promoting effect (Sun et al., 2021). It is speculated that efficient cellulose-degrading microbial strains probably play a vital role in the composting process. However, there is little research on the decomposition of cellulose during composting and the related cellulose-degrading microorganisms. It is of significance to isolate efficient cellulose-degrading indigenous microbial strains and utilize them as a consortium for rapid degradation of cellulose so as to provide technical support for the beneficial utilization of agricultural wastes with high cellulose contents.

In this study, seven efficient cellulose-degrading microbial strains were isolated from the compost samples using the Congo red staining test and the filter paper degradation test. The Congo red test mainly reflected the ability of the strains to degrade soluble cellulose such as CMC (Liang et al., 2014). In contrast, the filter paper degradation test reflected the stronger ability of the strains to degrade crystalline insoluble cellulose like filter paper (Guder and Krishna, 2019). Therefore, the strains screened based on the two tests had high cellulase activities. Of the seven strains, D-1 had the highest CMCase activity, A-2 strain had the highest FPase activity, and Z-1 strain had the highest β-Gase activity, indicating that different strains isolated from the same material can have very different activity patterns of the cellulase enzyme complex (Eida et al., 2012). Molecular identification of the strains showed that they were *T. reesei* (A-2), *Escherichia ferguson* (B-3), *Proteus vulgaris* (C-2), *Aspergillus griseus* (C-4), *Bacillus mycoides* (D-1), *C. glutamicum* (D-3), and *S. marcescens* (Z-1). Takuya et al. (2021) isolated a cellulase-producing *T. reesei* strain with β-Gase activity of 53.8 U/ml. Deena et al. (2021) found that *E. ferguson* could produce multiple degrading enzymes, had cellulose-degrading potential, and significantly reduced the dissolved solids in textile wastewater. Tan et al. (2019) showed that *P. vulgaris* secreted β-Gase, had cellulose-degrading ability, and grew well in the medium with cellulose as the sole carbon source. Chen et al. (2020) identified a GH5 cellulase, AgCMCase, from an *A. griseus* strain with activity of 291 U/ml after 4-days cultivation. The cellulase AgCMCase could degrade straw to produce reducing sugars with a yield of 1.61% when hydrolysis parameters had been optimized using RSM, indicating high cellulose-degrading capacity. Elisa and Rosa (2020) isolated a *B. mycoides* strain from an alpine forest soil with CMCase activity up to 1400 U/ml under optimized conditions. *C. glutamicum* can utilize various monosaccharides and organic matter as a carbon source to produce amino acids (Zhang et al., 2009). Sabrina et al. (2021) showed that *C. glutamicum* had cellulose-degrading potential. The present study also showed that *C. glutamicum* (D-3) had cellulase activity. Majumdar et al. (2020) showed that inoculation of *S. marcescens* to lignocellulose-rich paper mill sludge achieved a lignin degradation rate of 59% after 3 days. Zhou et al. (2021) isolated a *S. marcescens* strain from potato juice, which displayed a protein degradation rate of 61.43% under low temperature conditions, indicating that *S. marcescens* (Z-1) can not only degrade cellulose but also converse the rich protein in sesame oil cake.

It is commonly reported that microbial consortia composed of multiple strains present high cellulose-degrading capacities. Wang et al. (2015) designed a five-member microbial consortium, which showed significantly higher efficiency in degrading bitter ginseng residue and straw. Li J. et al. (2016) reported that the cellulose-degrading capacities of five microbial consortia were significantly improved (up to 50.71%) compared with their member strains. The antagonism test in this study showed that the seven strains were not antagonistic to each other and can be used to design microbial consortia. Eida et al. (2012) also reported a cellulose-degrading consortium, whose members were anaerobic and aerobic cellulose-degrading strains isolated from a compost pile. Consortia with random combinations of the seven strains were measured for their cellulase activities, and consortium VI, which was composed of all seven strains, had the highest cellulase activities. It is worth noting that in some cases, the cellulose-degrading capacity of a consortium is not only affected by its cellulase activity but also by its hemicellulase activity (Benedetti et al., 2019). This is because cellulose is often wrapped by a layer of hemicellulose (Gu et al., 2012), which keeps cellulose from the attack of cellulase, resulting in low efficiency of cellulose degradation. Therefore, consortia with both high activities of cellulase and hemicellulase are general more efficient in cellulose degradation. Studies have shown that fungal strains belonging to the genera *Aspergillus* and *Trichoderma* are the main hemicellulase-producing microorganisms (Mine et al., 2008). Li J. et al. (2016) reported that a consortium composed of three efficient cellulose-degrading bacterial strains had a straw degradation efficiency of merely 31.8%, indicating that consortia composed of only bacterial members may not have a desirable cellulose-degrading efficiency. Consortium VI in this study contained *T. reesei* (A-2) and *A. griseus* (C-4), which are known to have high hemicellulase activity (Gu et al., 2012; Xie, 2020). Therefore, when consortium VI was applied, hemicellulose would be degraded and cellulose would be exposed for efficient degradation by cellulase. This may explain the high efficiency of consortium VI in cellulose degradation. Future studies should focus more on candidate members with both high cellulase activity and high hemicellulase activity so as to further improve the efficiency of cellulose-degrading microbial consortia.

Cellulose-degrading microorganisms have been isolated from various materials and environments of different temperatures and pHs (Gong et al., 2020; Karthika et al., 2020; Yang et al., 2020). Shan et al. (2021) isolated a low temperature-tolerant cellulose-degrading bacterial strain from cow dung, with a cellulase activity of up to 130.21 U/ml at a culture temperature of 9°C. Jiang et al. (2021) obtained a high temperature-tolerant and efficient cellulose-degrading bacterial strain during the high-temperature stage of composting, whose relative enzyme activity was higher than 60% even at 85°C. These studies show that there are great differences in the suitable conditions for enzyme production of different strains. The strains in this study were isolated from samples taken at the heating, thermophilic, and maturity stages of composting, which had very different temperatures, pHs, and moisture contents. Therefore, the strains require different culture conditions for their optimal growth and enzyme production. When designing a microbial consortium to facilitate the composting of materials with high cellulose contents, heat-tolerant candidate members should be given the priority so that the consortium can work satisfactorily throughout the entire composting process. Response surface optimization based on single factor optimization is a common method to explore the optimal enzyme production conditions of microorganisms. Gong et al. (2020) used RSM to optimize the conditions for a three-member consortium, and straw weight was reduced by 60.55% after 7 days of composting. In this study, based on the results of single factor optimization experiments, RSM was employed to fine-tune the optimal conditions for the production of CMCase by consortium VI, and it was found that culture time had the greatest influence on CMCase production. In addition, under the conditions optimized for CMCase activity using RSM, the activities of FPase and β-Gase were also improved. Future studies should focus more on condition optimization for the activities of FPase and β-Gase so as to improve the overall cellulase production of consortia and promote the beneficial utilization of agricultural wastes with high cellulose contents.

The essence of composting is the physiological and biochemical process of microbial degradation of organic waste. During aerobic fermentation, the degradation rate of organic matter and CO_2_ release can reflect the composting fermentation speed and microbial metabolic intensity. Humus is a series of macromolecular substances of different molecular weights and oxygen-containing functional groups produced by the interaction of microorganisms and enzymes, which represents the degree of pile humification. The results of this experiment showed that consortium VI could significantly improve the fermentation efficiency of compost and promote the process of humification. This may be that cellulose-degrading strains are conducive to the degradation of lignocellulose and other substances, improve fermentation parameters, and promote the formation of humic acid precursors. Similar results could be observed on the compost of chicken feces inoculated with cellulose degrading strains (Li S. Y. et al., 2016). Nitrogen is a necessary element for microbial metabolism and biosynthesis, and it is also an important nutrient index for composting product quality. C/N ratio and GI are the important indicators of compost maturity and the biological toxicity of compost products. The results indicated that the cellulose degrading consortium VI inoculation resulted in higher total nitrogen content, faster maturity and lower biological toxicity of the compost. This may be that cellulose-degrading strains enhanced the “concentration effect” and “ammonia assimilation effect,” nitrogen loss and C/N ratio were reduced. Gou et al. (2017) reported that inoculation with cellulose-degrading bacteria during rice straw composting could significantly reduce C/N ratio and increase GI value. Cellulose strains increased the content of active substances in compost, and had better growth promoting effect, which was consistent with the results of this study.

## Conclusion

In this study, cellulose-degrading microbial strains were isolated from compost samples taken from different composting stages and further screened using the Congo-red staining method and filter paper degradation test. Seven strains with high activities of CMCase, FPase, and β-Gase were obtained. They are *T. reesei*, *E. fergusonii*, *P. vulgaris*, *A. glaucus*, *B. mycoides*, *C. glutamicum*, and *S. marcescens.* Six microbial consortia were designed with these strains. Compared with the other five consortia, consortium VI composed of all seven strains displayed the highest activities of CMCase, FPase, and β-Gase, 141.89, 104.56, and 131.18 U/ml, respectively. Single factor approach was first employed to optimize the CMCase production of consortium VI, and then RSM was employed to fine-tune the culture conditions for optimal CMCase production. The optimal conditions for CMCase production of consortium VI were: culture time 4.25 days, culture temperature 35.5°C, pH 6.6, and inoculum volume 5% (v/v). Under the optimized conditions, the CMCase activity of consortium VI was 170.83 U/ml. Compared with EM microbial agent, consortium VI showed better effect in aerobic fermentation of rabbit feces and sesame oil cake. In this treatment, the organic matter degradation was faster, the total nitrogen and humic acid content were increased more, and the maturity of the pile were improved. This work provides a technical basis for cellulose-degrading microbial consortia design, which is expected to help facilitate the composting of materials with high cellulose contents. This work is of significance in that it will promote the beneficial utilization of agricultural wastes and greener development of agriculture.

## Data availability statement

The original contributions presented in the study are included in the article/Supplementary material, further inquiries can be directed to the corresponding author.

## Author contributions

YD: conceptualization, methodology, writing–review and editing, and supervision. GZ: data curation, writing–original draft preparation, software, and investigation. Both authors contributed to the article and approved the submitted version.

## Conflict of interest

The authors declare that the research was conducted in the absence of any commercial or financial relationships that could be construed as a potential conflict of interest.

## Publisher’s note

All claims expressed in this article are solely those of the authors and do not necessarily represent those of their affiliated organizations, or those of the publisher, the editors and the reviewers. Any product that may be evaluated in this article, or claim that may be made by its manufacturer, is not guaranteed or endorsed by the publisher.

## References

[B1] Agrawal R. Verma A. Singhania R. R. Varjani S. Di Dong C. Kumar Patel A. (2021). Current understanding of the inhibition factors and their mechanism of action for the lignocellulosic biomass hydrolysis. *Bioresour. Technol.* 332:25042. 10.1016/j.biortech.2021.125042 33813178

[B2] Agrawal R. Verma A. K. Satlewal A. (2016). Application of nanoparticle-immobilized thermostable β-glucosidase for improving the sugarcane juice properties. *Innov. Food Sci. Emerg. Technol.* 33 472–482. 10.1016/j.ifset.2015.11.024

[B3] Benedetti M. Vecchi V. Betterle N. Natali A. Bassi R. Osto L. (2019). Design of a highly thermostable hemicellulose-degrading blend from *Thermotoga neapolitana* for the treatment of lignocellulosic biomass. *J. Biotechnol.* 296 42–52. 10.1016/j.jbiotec.2019.03.005 30885654

[B4] Boddy L. (2000). Interspecific combative interactions between wood-decaying basidiomycetes. *FEMS Microbiol. Ecol.* 31 185–194. 10.1016/S0168-6496(99)00093-8 10719199

[B5] Bu K. N. Ji B. S. (1984). *Bergey’s Manual of Determinative Bacteriology.* Beijing: Science Press

[B6] Chen L. Wei Y. Shi M. Li Z. Zhang S. H. (2020). Statistical optimization of a cellulase from *Aspergillus glaucus* CCHA for hydrolyzing corn and rice straw by RSM to enhance yield of reducing sugar. *Biotechnol. Lett.* 42 583–595. 10.1007/s10529-020-02804-5 31980972

[B7] Cheng Y. (2017). *Studies on Degraded Conditions of Sesame Cake and its Influential Factors*, Ph.D thesis, Hunan: Agricultural University

[B8] Deena S. R. Varsha N. S. Thiagarajan R. Ponnusami V. Meera P. (2021). Remediation of textile effluents for water reuse: decolorization and desalination using *Escherichia fergusonii* followed by detoxification with activated charcoal. *J. Environ. Manage.* 277:111406. 10.1016/j.jenvman.2020.111406 33038672

[B9] Dong X. Y. Zhan P. Y. Ren X. Q. Hu H. H. Tang S. Q. (2017). Isolation, screening and identification of cellulase producing bacteria from rabbit manure. *J. Hebei Agric. Sci.* 21 56–59. 10.16318/j.cnki.hbnykx.2017.05.013

[B10] Dong X. Z. Cai M. Y. (2001). *Manual of Identification of Common Bacterial Systems.* Beijing: Science Press.

[B11] Eida M. F. Nagaoka T. Wasaki J. Kouno K. (2012). Isolation and characterization of cellulose-decomposing bacteria inhabiting sawdust and coffee residue composts. *Microbes Environ.* 27 226–233. 10.1264/jsme2.ME11299 22353767 PMC4036048

[B12] Elisa S. Rosa M. (2020). Production and partial characterization of a crude cold-active cellulase (CMCase) from *Bacillus mycoides* AR20-61 isolated from an Alpine Forest site. *Ann. Microbiol.* 70:67. 10.1186/s13213-020-01607-3

[B13] Gong X. Zou H. Qian C. Yu Y. Hao Y. Li L. (2020). Construction of in situ degradation bacteria of corn straw and analysis of its degradation efficiency. *Ann. Microbiol.* 70:62. 10.1186/s13213-020-01601-9

[B14] Gou C. Wang Y. Zhang X. Lou Y. Gao Y. (2017). Inoculation with a psychrotrophic-thermophilic complex microbial agent accelerates onset and promotes maturity of dairy manure-rice straw composting under cold climate conditions. *Bioresour. Technol*. 243 339–346. 10.1016/j.biortech.2017.06.097 28683387

[B15] Gu W. J. Xu Y. Q. Xu P. Z. Xie K. Z. Lu Y. S. Tang S. H. (2012). Screening and identification of hemicellulose degrading microorganisms in acid soil. *Acta Microbiol. Sin.* 52 1251–1259. 10.13343/j.cnki.wsxb.2012.10.004 23289324

[B16] Guder D. G. Krishna M. S. R. (2019). Isolation and characterization of potential cellulose degrading bacteria from sheep rumen. *J. Pure Appl. Microbiol*. 13 1831–1839. 10.22207/JPAM.13.3.60

[B17] Jiang G. F. Yang T. J. Zheng H. P. Wei Z. Wang S. M. Fan X. T. (2021). Construction and evaluation of fungal consortia effect on maize straw degradation. *J. Plant Nutr. Fertil*. 27 284–292. 10.11674/zwyf.20363

[B18] Karthika A. Seenivasagan R. Kasimani R. Babalola O. O. Vasanthy M. (2020). Cellulolytic bacteria isolation, screening and optimization of enzyme production from vermicompost of paper cup waste. *Waste Manag.* 116 58–65. 10.1016/j.wasman.2020.06.036 32784122

[B19] Kaur P. Sharma N. Munagala M. Rajkhowa R. Aallardyce B. Shastri Y. (2021). Nanocellulose: resources, physio-chemical properties, current uses and future applications. *Front. Nanotechnol.* 3:747329. 10.3389/fnano.2021.747329

[B20] Kjeldahl J. (1883). Neue Methode zur Bestimmung des Stickstoffs in organischen Körpern. *Zeitschrift für Analytische Chemie* 22 366–382.

[B21] Kurtzman C. P. Robnett C. J. (1997). Identification of clinically important ascomycetous yeasts based on nucleotide divergence in the 5’ end of the large-subunit (26S) ribosomal DNA gene. *J. Clin. Microbiol.* 35 1216–1223. 10.1128/jcm.35.5.1216-1223.1997 9114410 PMC232732

[B22] Lane D. J. (1991). “16S/23S rRNA Sequencing,” in *Nucleic Acid Techniques in Bacterial Systematics*, eds Stackebrandt E. Goodfellow M. (New York, NY: John Wiley and Sons).

[B23] Li J. Zhang H. N. Zhao C. Zhang J. Y. Zhang Q. Zhang J. Y. (2016). Isolation and screening of cellulose decomposing microbe and the straw decomposing effect of complex microbial system. *Chin. J. Appl. Environ. Biol*. 22 0689–0696. 10.3724/SP.J.1145.2015.10040

[B24] Li S. Y. Li J. J. Zhang B. X. Li G. X. Li Y. Y. Li D. Y. (2016). Influence of inoculants on content and quality of humus during chicken manure composting. *Trans. Chin. Soc. Agric. Eng.* 32 268–274. 10.11975/j.issn.1002-6819.2016.z2.037

[B25] Li Y. J. Liu L. C. Yang B. Zhang W. D. Yin F. Xu L. (2013). Experimental study on biogas production by mesophilic fermentation for rabbit dung. *Adv. Mat. Res.* 763 160–164. 10.4028/www.scientific.net/AMR.763.160

[B26] Liang Y. L. Zhang Z. Wu M. Wu Y. Feng J. X. (2014). Isolation, screening, and identification of cellulolytic bacteria from natural reserves in the subtropical region of China and optimization of cellulase production by *Paenibacillus terrae* ME27-1. *Biomed. Res. Int*. 2014:512497. 10.1155/2014/512497 25050355 PMC4090499

[B27] Liu X. (2019). *Construction and Effectiveness of a High Efficient Complex Microbial System for Corn Straw Degradation*, Ph.D thesis, Dongbei: Agricultural University.

[B28] Majumdar S. Paul I. Dey S. Dutta S. Mandal T. Mandal D. D. (2020). Biotransformation of paper mill sludge by *Serratia marcescens* NITDPER1 for prodigiosin and cellulose nanocrystals: a strategic valorization approach. *Biochem. Eng. J.* 164:107766. 10.1016/j.bej.2020.107766

[B29] Mao T. Wei Y. Q. Yang H. J. Niu Y. Y. Chen J. Wang Z. Y. (2019). Screening of celluloytic bacterium from yak faeces and the optimization of cellulase production conditions. *J. Agric. Univ*. 24 106–116. 10.11841/j.issn.1007-4333.2019.11.12

[B30] Mesa I. M. Situmeang Y. P. Wirajaya A. A. N. M. Udayana I. G. B. Yuliartini M. S. (2021). Utilization of rabbit manure and biochar chicken manure and its effect on the growth and yield of pakchoy plants. *J. Phys. Conf. Ser.* 1869:012045. 10.1088/1742-6596/1869/1/012045

[B31] Mine H. K. Keiko T. Yasuki F. Wataru H. Kousaku M. (2008). *Paenibacillus* sp. strain HC1 xylanases responsible for degradation of rice bran hemicellulose. *Microbiol. Res.* 163 293–298. 10.1016/j.micres.2006.05.011 16829064

[B32] Montes-Carreto L. M. Aguirre-Noyola J. L. Solis-Garcia I. A. Ortega J. Martinez-Romero E. Guerrero J. A. (2021). Diverse methanogens, bacteria and tannase genes in the feces of the endangered volcano rabbit (Romerolagus diazi). *PeerJ*. 9:e11942. 10.7717/peerj.11942 34458021 PMC8378336

[B33] Rawway M. Ali S. G. Badawy A. S. (2018). Isolation and identification of cellulose degrading bacteria from different sources at Assiut Governorate (Upper Egypt). *J. Ecol. Health Environ*. 6 15–24. 10.18576/jehe/060103

[B34] Sabrina W. Judith B. Yota T. Hideo K. Akihiko K. Jan M. (2021). Advances in metabolic engineering of *Corynebacterium glutamicum* to produce high-value active ingredients for food, feed, human health, and well-being. *Essays Biochem.* 65 197–212. 10.1042/EBC20200134 34096577 PMC8313993

[B35] Shaikh N. M. Patel A. A. Mehta S. A. Patel N. D. (2013). Isolation and screening of cellulolytic bacteria inhabiting different environment and optimization of cellulase production. *Univers. J. Environ. Res. Technol.* 3 39–49. 10.5897/AJBM11.2824

[B36] Shan J. R. Quan X. Zhu Y. Z. Xing Y. Zhang X. Wang H. Y. (2021). Screening of a low-temperature cellulose-degrading bacterium and optimization of cellulase production conditions. *Chin. J. Plant Ecol.* 40 1128–1136. 10.13292/j.1000-4890.202104.005

[B37] Sun G. Y. Zhang G. Y. Dong Y. J. (2021). Composting process of agricultural wastes from different sources and fertilizer efficiency of their products. *J. Soil Water Conserv*. 35 349–360. 10.13870/j.cnki.stbcxb.2021.04.048

[B38] Takuya N. Haruka S. Ryuji N. Naritoshi Y. Tsuneo M. Yoshiko T. (2021). Isolation of a cellulase hyperproducing mutant strain of *Trichoderma reesei*. *Bioresour. Technol. Rep.* 15:100733. 10.1016/j.biteb.2021.100733

[B39] Tan W. Q. Li R. Wang N. Wang H. X. Jiang L. F. Jiang Q. W. (2019). Study on the biological characteristics of *Proteus vulgaris* and its ability to utilize dietary fiber in vitro. *Chin. J. Health Lab. Tec.* 29 897–900.

[B40] Tuli D. K. Agrawal R. Satlewal A. Mathur A. S. Gupta R. P. Raj T. (2018). Kinetic and enzyme recycling studies of immobilized β-glucosidase for lignocellulosic biomass hydrolysis. *Environ. Eng. Manag J.* 17 1385–1398. 10.30638/eemj.2018.137

[B41] Wang H. B. Han L. R. Feng J. T. Zhang X. (2015). Screening of highly efficient cellulose degradation microbes and construction of composite strains. *J. Agric. Biotechnol*. 23 421–431. 10.3969/j.issn.1674-7968.2015.04.001

[B42] Wang X. H. Xu X. Shan Q. M. K. Wang H. Ye K. Li G. (2018). Optimization of pretreatment process for corn straw anaerobic digest. *Trans. Chin. Soc. Agric. Eng.* 34 246–253. 10.11975/j.issn.1002-6819.2018.23.032

[B43] Xi B. He X. Dang Q. Yang T. Li M. Wang X. (2015). Effect of multi-stage inoculation on the bacterial and fungal community structure during organic municipal solid wastes composting. *Bioresour. Technol.* 196 399–405. 10.1016/j.biortech.2015.07.069 26257051

[B44] Xie X. (2020). *Effect of Sec6 Gene of Trichoderma Reesei on Mycelial Branching and Enzyme Production*, Ph.D thesis, Henan: Agricultural University, 10.27117/d.cnki.ghenu.2020.000020

[B45] Yang G. Yang D. Q. Cao W. T. Wang X. D. (2020). Screening of cellulose degrading bacteria in distiller’s grains and degradation technology of distiller’s grains. *Trans. Chin. Soc. Agric. Eng*. 36 212–221. 10.11975/j.issn.1002-6819.2020.13.025

[B46] Zhang S. Shan D. Liu X. Sun M. (2018). Cellulose-degrading strains: their screening and application to corn straw in low-temperature environments. *Pol. J. Environ. Stud.* 27 2349–2355. 10.15244/pjoes/79272

[B47] Zhang X. M. Dou W. F. Xu H. Y. Xu Z. H. (2009). Identification of L-Serine producer SYPS-062 and the effect of different carbon source. *Microbiol. Chin*. 36 789–793. 10.13344/j.microbiol.china.2009.06.001

[B48] Zhou H. N. Zhang Y. M. Zhang H. L. Li H. F. (2021). Screening of a strain degrading protein in the juice of potato starch and analysis of its fermentation products. *Food Fermentation Industries* 47 158–164. 10.13995/j.cnki.11-1802/ts.025776

