# Supplementary figures and images for: Design and application of an efficient cellulose-degrading microbial consortium and carboxymethyl cellulase production optimization
Source: Front Microbiol. 2022 Jul 15;13:957444. doi: 10.3389/fmicb.2022.957444 (PMC9335055; doi:10.3389/fmicb.2022.957444)

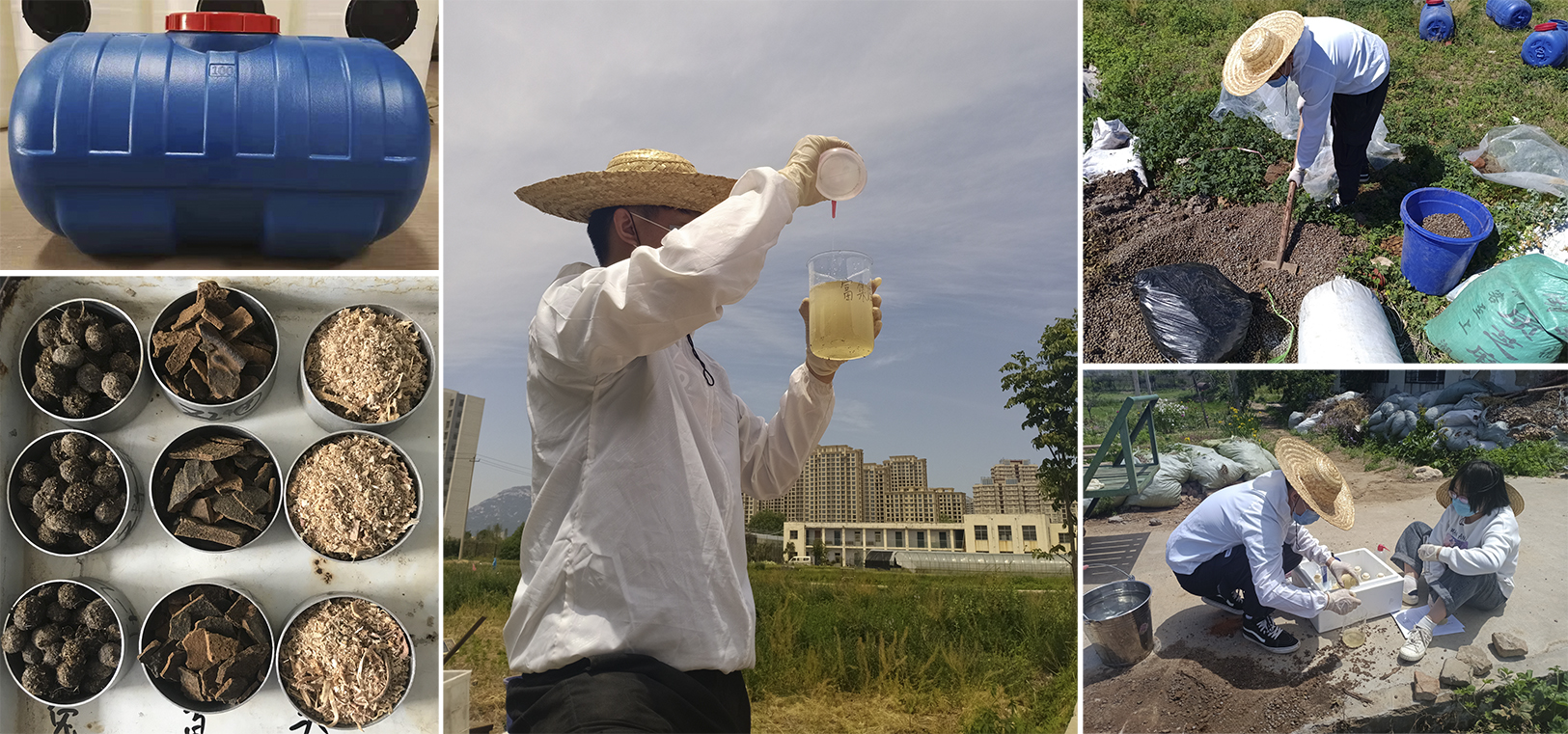

Supplement: Supplementary file 1 [file Image_1.JPEG]

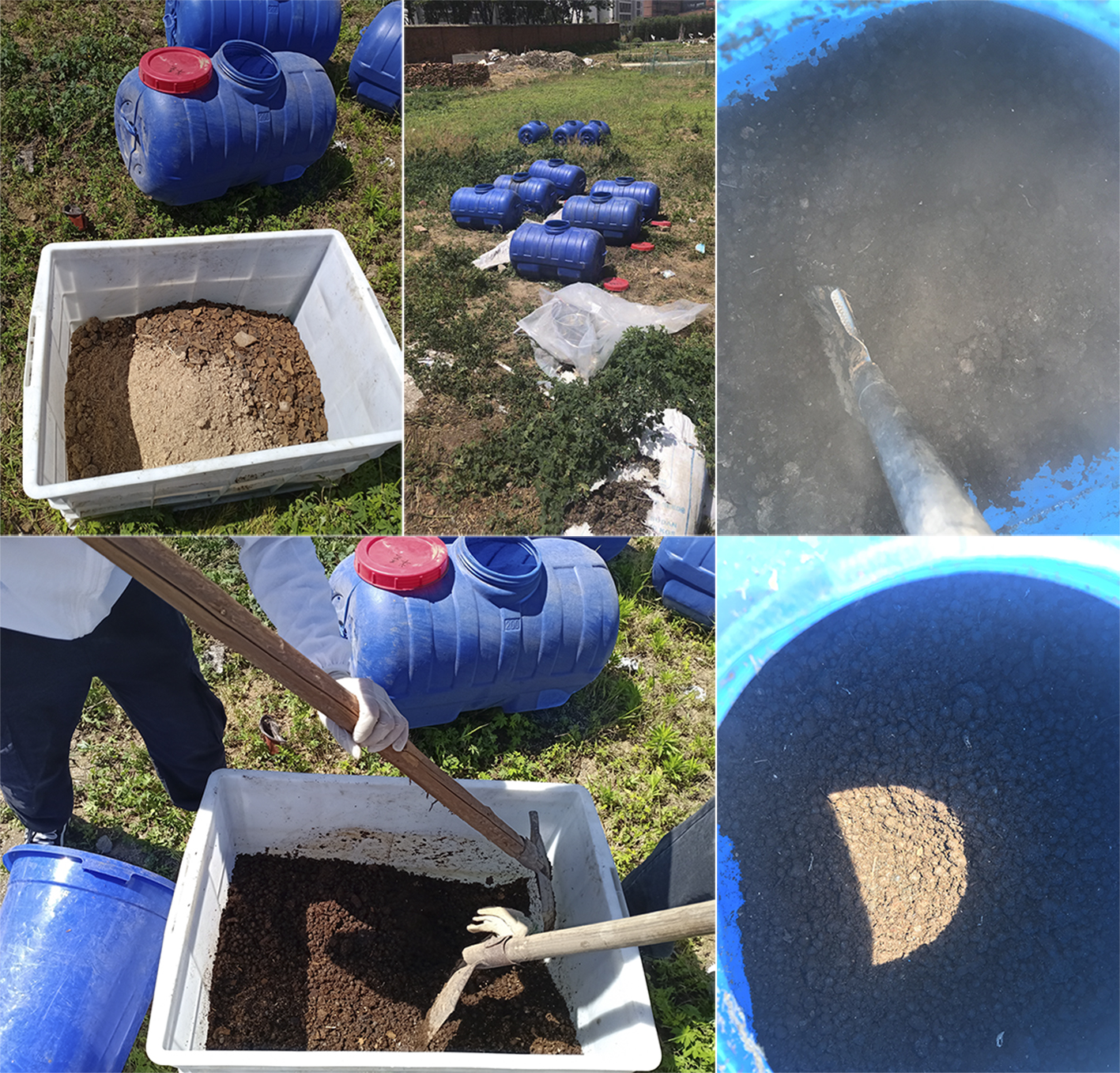

Supplement: Supplementary file 2 [file Image_2.JPEG]
